# Transgenic Models of Spinocerebellar Ataxia Type 10: Modeling a Repeat Expansion Disorder

**DOI:** 10.3390/genes3030481

**Published:** 2012-07-30

**Authors:** Karen N. McFarland, Tetsuo Ashizawa

**Affiliations:** Department of Neurology, The McKnight Brain Institute, University of Florida, Gainesville, FL 32610, USA; E-Mail: tetsuo.ashizawa@neurology.ufl.edu

**Keywords:** RNA-mediated gain-of-function, genotype-phenotype correlations, autosomal dominant cerebellar ataxia, repeat expansion

## Abstract

Spinocerebellar ataxia type 10 (SCA10) is an autosomal dominant neurodegenerative disease with a spectrum of phenotypes. SCA10 is caused by a pentanucleotide repeat expansion of the ATTCT motif within intron 9 of *ATAXIN 10 (ATXN10)*. Patients present with cerebellar ataxia; however, a subset also develops epileptic seizures which significantly contribute to the morbidity and mortality of the disease. Past research from our lab has demonstrated that epileptic SCA10 patients predominantly originate from or have ancestral ties to Mexico. In addition, a large proportion of epileptic SCA10 patients carry repeat interruptions within their SCA10 expansion. This paper outlines the variability in SCA10 phenotypes and our attempts to model these phenotypes using transgenic mouse models and highlights the benefits of using a transgenic model organism to understand the pathological mechanisms of a human disease.

## 1. Introduction

### 1.1. SCA10—An Autosomal Dominant, Neurodegenerative Disorder

Spinocerebellar ataxia type 10 (SCA10 [MIM ID #603516]) is an autosomal dominant, neurodegenerative disorder. Progressive ataxia and anticipation are typical disease features and patients sometimes present with epileptic seizures. SCA10 is the second most common form of autosomal dominant cerebellar ataxia in Mexico and Brazil after SCA2 and SCA3, respectively [1] and has been found in patients from Central and South American populations with Amerindian ancestry.

SCA10 results from the expansion of a noncoding pentanucleotide repeat (ATTCT) within intron 9 of the *Ataxin 10* (*ATXN10*) gene on 22q13 [2]. While the ATTCT repeat normally ranges from 10 to 29 repeats, the disease allele size expands up to 4,500 repeats in length and intermediate alleles with incomplete penetrance have been found varying from 280 to 850 repeats [2,3,4].

### 1.2. Phenotypic Variability of SCA10

Disease phenotypes can vary amongst SCA10 patient populations and typically correlate with the patient’s geographic origin [5]. Disease onset ranges from 12–48 years of age. Initial disease symptoms invariably consist of poor balance with an unsteady gait, followed by other cerebellar symptoms such as scanning dysarthria and dysphagia, involuntary eye movements such as irregular pursuit and nystagmus and uncoordinated movements of intended actions, such as dysmetria, intention tremor and dysdiadochokinesia [6,7,8,9]. The incidence of epilepsy in SCA10 families is perhaps the most striking phenotypic variable. Seizure onset occurs after the onset of cerebellar ataxia, usually with a gap of about five years [6,7]. Seizures in these patients can be generalized motor and/or complex partial seizures with occasional secondary generalization [6,7]. Seizure symptoms in SCA10 patients can usually be controlled by anticonvulsant medications provided that patients are compliant [6]. However, intractable epileptic seizures are a significant contributing factor to the morbidity and mortality of SCA10 [6].

The incidence of epilepsy is up to 80% within SCA10 Mexican families although some Mexican families have no history of epilepsy [6,7,10]. Two SCA10 patients from Argentina also presented with epilepsy [9], as did a lone Venezuelan SCA10 patient [11]. Conversely, in Brazilian SCA10 families, the frequency of epilepsy is much lower (3.75%) [12]. What factors are present that cause some SCA10 patients to develop epilepsy?

## 2. Repeat Purity as a Phenotypic Modifier?

Sequencing a short, 280 repeat SCA10 allele of reduced penetrance revealed ATGCT sequence interruptions within the ATTCT expansion and prompted attempts at sequencing into the interior of longer SCA10 expansions [3]. These experiments revealed ATATTCT and ATTTTCT heptanucleotide interruptions at the 5' end of the repeat from one Mexican SCA10 family with a high incidence of epilepsy while a second family with a lower incidence of epilepsy lacked these interruptions [3]. These data lead to the hypothesis that repeat purity plays a significant role in phenotypic expression of SCA10. However, the limitations of current sequencing technologies, including next generation sequencing, combined with the intrinsic nature of the repetitive sequence prevents us from sequencing across the entirety of the expansion.

## 3. SCA10 as an RNA Mediated Gain-of-Function Mutation

*ATXN10* mRNA and protein are both widely expressed throughout the body as well as the brain [2,13,14]. *ATXN10* encodes a 52 kDa protein whose features are rather unremarkable with the exception of two C-terminal armadillo repeats and stretches of proposed alpha-helices [13].

ATXN10 protein is required for neuronal survival [15] and can induce neurite outgrowth when overexpressed [14]. ATXN10 interaction partners include the G-protein beta 2 subunit (Gbeta2) involved in signaling cascades [16] and *O*-linked *N*-acetylglucosamine transferase [17,18].

At the DNA structural level, the ATTCT repeats cause an unpaired DNA structure when present within a supercoiled plasmid and acts as a DNA unwinding element (DUE) in *E. coli* [19] which may help to explain how the expansion can function as a replication origin initiator in mammalian cell extracts [20]. Errors during the replication process or aberrant replication starting may play a role in repeat instability and fragility of the SCA10 expansion as demonstrated in a yeast model system expressing ATTCT repeats within the context of an artificially constructed intron within the URA3 gene [21]. Additionally, *in vitro* assays of nucleosome formation suggest that SCA10 repeats sequences can readily form nucleosomes [22]. Combined, these studies further suggest that the SCA10 expansion itself may have an effect on the local chromosomal structure and may assert effects on the local chromosomal environment in *cis*.

However, SCA10 patient phenotypes are not likely to result from a loss of function of the *ATXN10* gene product. *ATXN10* transcript levels from SCA10 patients are similar to normal controls and *Atxn10* heterozygous knockout mice are morphologically and behaviorally normal despite reduced *Atxn10* levels [14]. Furthermore, there are no signs of ataxia or epilepsy in the members of a family carrying the translocation t(2,22) (p25.3; q13.31) which disrupts one *ATXN10* copy [23].

During pre-mRNA processing, the intron bearing expanded SCA10 repeats is spliced out and accumulates as AUUCU-containing cytoplasmic and nuclear foci [24,25]. Short, non-pathogenic lengths of AUUCU repeats take on a hairpin structure although pathogenic lengths of SCA10 expansions have not been tested [26]. The RNA binding protein, hnRNP K, was identified as an AUUCU interacting protein by pull-down assay using protein extracts from mouse brains and was later found colocalized with AUUCU RNA foci within SCA10 patient fibroblasts and transgenic mouse brains [24,25].

## 4. Prior Animal Models of SCA10

Questions regarding phenotypic variability in SCA10 can be modeled in transgenic mice. However, the transgenic mouse lines studied in the past are informative yet limiting for reasons described below. Because of these limitations, we have taken this opportunity to reexamine these models and how they might be improved. Additionally, both SCA10 transgenic models were made using SCA10 expansions from patients carrying repeat interruptions and as the corresponding transgenic model with pure SCA10 repeats was not constructed, a number of questions still remain regarding the impact of repeat purity on disease progression, ataxia and epileptic phenotypes.

### 4.1. SCA10 Intronic Model

The first transgenic SCA10 mouse model was published in 2010 [24]. Here, an interrupted SCA10 repeat expansion was cloned from a SCA10 somatic cell hybrid line and inserted within a rabbit*-globin* intron located upstream of the *LacZ* reporter gene. This transgene was placed under the control of the rat *neuronal enolase* (*Eno2*) promoter. Mice carrying the transgene recapitulate a number of the same molecular phenotypes seen in fibroblast cell lines obtained from SCA10 patients (Table 1). By 6 months, mice develop AUUCU-containing RNA foci that colocalize with hnRNP K protein. There is also mis-splicing of hnRNP K target genes as well as an increase of PKCδ mislocalization to the mitochondria suggesting that the sequestration of hnRNP K to RNA foci prevents it normal functions of acting as a splicing regulator and partnering with PKCδ and preventing its translocation to the mitochondria where it sets off the apoptotic cascade. 

Importantly, these mice demonstrate that the expression of the non-coding SCA10 repeat expansion is sufficient to produce molecular phenotypes similar to those seen in SCA10 and validates the pathogenic mechanism as one of an RNA-mediated gain of function. Unfortunately, these mouse lines breed poorly making them difficult to work with.

**Table 1:** Comparison of Molecular Analyses of SCA10 transgenic mouse lines.

		SCA10 patient fibroblasts	Transgenic Mice (*Eno2*-β-globin intron-LacZ)	Transgenic Mice (*Prnp*-LacZ-3'UTR)
	Mouse Strain	n.a.	C57/Bl/6	FVB/N
	Repeat Expansion	1,000 repeats	500 repeats	500 repeats
Molecular Phenotypes	LacZ mRNA (via qRT-PCR)	n.a.	n.d.	Throughout brain including cerebellum, also outside of brain
	**β**-gal protein (via immunostaining)	n.a.	n.d.	Lowest in Purkinje Cells of cerebellum, highest in cerebral cortex and pontine nuclei
	RNA foci (via FISH)	Yes	Yes, at 3 mon & 6 mon in cortex	Yes, at 3 & 6 months in cortex, pontine and hippocampusNot found at 1 month
	hnRNP K/RNA foci colocalization (via FISH/immunostaining)	Yes	Yes, at 3 mon & 6 mon in cortex	Yes at 6 months in pontine nuclei & hippocampus
	Apoptosis (via TUNEL)	n.d.	n.d.	None in hippocampal CA3
	Gliosis? (via GFAP immunostaining)	n.a.	n.d.	None in hippocampal CA3
	Splicing Defects of hnRNP K target exons	Increased use of exon 6A vs exon 6B in *TPM2*	n.d.	n.d.
	PKCδ mitochondrial localization (via colocalization &/or fractionation)	Increased	Increased	Increased
	hnRNPK-PKCδ interactions (via co-IP)	Decreased	n.d.	n.d.
Pathological phenotypes	Neuropathological	n.a.	n.d.	Neuronal loss in hippocampal CA3 region; no obvious neuronal loss in cerebellum
	Other findings	n.a.	n.d.	Increased glycogen accumulation in frontal lobe starting at 3 months, increasing by 6 months, nearly 100% of neurons by 18 months
Behavioral Phenotypes	Open Field at 6 months	n.a.	n.d.	Traveled lesser distance and at slower speed with fewer crossings
	Footprint Analysis at 6 months	n.a.	n.d.	Step length shortened with step width variability and awkward hindlimb movements
	Rotarod, fixed speed at 6 months	n.a.	n.d.	normal
	Rotarod, accelerated at 6 months	n.a.	n.d.	normal
	Hindlimb Clasp at 3 & 6 months	n.a.	n.d.	Yes, all by 6 months
				About half by 3 months
	Other, abnormal at 6 months	n.a.	Decreased reproductive fitness	Decreased grooming, abnormal whisker twitch reflex
	Other, Normal at 6 months	n.a.	n.d.	Eye blink, ear twitch & righting reflexes
	Home Cage Behavior	n.a.	n.d.	Demonstrate preconvulsant behaviors
	Seizure susceptibility	n.a.	n.d.	Seizures at low doses of PTZ; full tonic-clonic seizures and death at higher doses

### 4.2. SCA10 3' UTR Model

At the same time, a second transgenic model was developed. For this model, the SCA10 repeat expansion, cloned from SCA10 hybrid cell lines with an interrupted SCA10 expansion, was inserted into the bovine growth hormone 3' untranslated region (UTR) and polyadenylation (PolyA) tail located downstream of the LacZ transgene [25]. Expression of the transgene is driven by the *prion* (*Prnp*) promoter which is sufficient for expression throughout a large portion of the brain [27]. Although this model is not a faithful recapitulation of the disease where the repeat is present within the intron, the resulting molecular and behavioral phenotypes that result from a non-coding SCA10 repeat expansion recapitulate that non-coding AUUCU RNA expansions are the toxic entity in SCA10 pathogenesis.

Like the SCA10 intron model, RNA foci containing the SCA10 expansion are formed by 6 months and can be detected as early as 3 months of age. The RNA foci contain the RNA-binding protein hnRNP K. Neurodegeneration in these mice is present in the hippocampus but not the cerebellum, the site of atrophy in SCA10 patients. However, this difference in pathology may be accounted for by the use of the *Prnp* promoter and not the native *ATXN10* promoter. The neuronal loss appears to result not from apoptosis or gliosis but rather from glycogen accumulation. Strikingly, these mice develop gait abnormalities and dyskinetic movements (hindlimb clasping) indicating motor problems. In addition, these mice display preconvulsant behaviors in their home cage and are more sensitive to drug-induced seizures. However, this line of mice was lost due to Hurricane Ike which made landfall near Galveston, TX in September 2008. 

## 5. Improvements for the Future

Both the intron and the 3'UTR models clearly demonstrate the role of the non-coding repeat expansion as the toxic agent in the pathogenic progression of SCA10. However, due to the loss of one model and the poor reproductive capacity of the other, new SCA10 models need to be made. At this time, we need to reassess what is required in order to make the best model possible.

First, in order to understand the role of repeat interruption and repeat purity on phenotypic expression of SCA10, transgenic models that expression interrupted and non-interrupted versions of the SCA10 expansion need to be developed and compared. As transgenic constructs randomly integrate into the genome, transgenic lines will need to be carefully matched for transgene copy number and expression levels in order to have comparable lines. Alternatively, targeted transgenic lines where the transgene is selectively targeted into a known genomic locus, such as the ROSA26 locus [28] could be an option; however, the stability of the SCA10 expansion in embryonic stem cells is unknown at this time.

A second consideration is the genetic background used for making transgenic mice. This can matter a great deal for phenotypic expression, particularly in our prior intronic SCA10 model. As these mice breed poorly and were made on a C57/Bl6 background, it leaves us to wonder whether there is a connection between the two. The choice in genetic background is one of great impact as sensitivity to epileptic phenotypes are known to vary across the different background strains [29] and some strains are more sensitive to drug-induced seizures, such as the DBA/2J strain [30], while the C57Bl/6 strain are relatively more resistant and display little to no excitotoxic cell death [31].

Another point to consider lies in the choice of reporter gene used in the expression cassette with the SCA10 expansions. The reporter gene ideally should be one that is robust and easy to assay. Obvious choices include *green fluorescent protein* (GFP), or a fluorescent protein of another variety, *luciferase* or *LacZ*. GFP carries the obvious advantage as it can be visualized in the live animal; however, luciferase is more useful for sensitive, quantifiable applications. In addition, there should be a method in place to easily assess correct splicing of the intron containing the SCA10 expansion.

Another important consideration is the choice in promoter used to drive expression of the transgene construct. Ideally the endogenous *ATXN10* promoter would give the most faithful recapitulation of the native ATXN10 expression pattern. However, the minimal *ATXN10* promoter, from any species, has yet to be defined. Alternatively, a BAC construct carrying the SCA10 expansions could be made from human SCA10 patients. Such a construct would contain endogenous human promoter elements making it an attractive alternative. However, after attempts with two BAC libraries made from SCA10 patient genomic DNA, we were unable to isolate such a BAC clone (unpublished data). This leaves us with the choice of using neuronal-specific promoters to drive expression within the brain or certain subsets of neuronal population or using an inducible promoter allowing us to control the timing and location of expression.

The choice of which promoter to use may prove to be a crucial one as we are currently unsure of the extent of neuronal populations that are affected in SCA10 pathology given the relative dearth of autopsy tissue available for analysis. We do know that the cerebellum and sometimes the brainstem show signs of atrophy by imaging analysis of SCA10 patients [6,7,12] therefore the transgene should be expressed in these regions at a minimum. Thus, promoters to consider should be either pan-neuronal, such as *neuronal enolase* (*Eno2*) promoter [32] as was used in the intronic SCA10 model [24] or a cell-type restrictive, such as *Purkinje cell protein-2 (Pcp2)*, promoter which drives expression in the Purkinje cells of the cerebellum [33,34].

However, an inducible expression system, such as the bi-transgenic tet-operon/repressor system (reviewed in [35]), might have an advantage over conventional transgenic models as precise control of tissue- and timing-specific expression is gained with this system. The tetracycline transactivator (tTA) protein in the transactivator transgene, is made up of the tet-repressor protein from the *E. coli* Tn10 transposon fused to the VP16 transactivation domain from herpes simplex virus [36]. The responder construct contains the expression of the transgene of interest downstream of the Tc response element (TRE) consisting of the minimal cytomegalovirus (CMV) promoter with seven tandem repeats of the 17 bp *tet* operator (*tetO*). Binding of the dimerized tTA protein at the *tet*O sites causes expression of the transgene in the absence of a tetracycline analog and turns off expression in the presence of a tetracycline analog (Tet-Off). Conversely, an engineered reverse tetracycline transactivator (rtTA) protein allows for expression of the transgene in the presence of tetracycline (Tet-On) for easier control in the timing of the transgene expression in the mouse [37].

## 6. Rigorous Assessment to Compare Behavioral Phenotypes

Whatever the mode of transgenic model production, the resulting lines of mice will need to be rigorously assessed for deficiencies in behavioral, movement and seizures susceptibility. Well-known batteries of behavioral assays have been described by the Irwin [38] and SHIRPA [39] protocols as well as in numerous behavioral and neurological phenotyping guides [40,41,42,43]. The chosen assays should be simple and easy enough to rapidly perform without the need for specialized equipment and yet also allow for quantification of results. Results of an initial battery of tests can then be used to guide more detailed, intensive behavior phenotyping.

## 7. Conclusions

The process of developing a transgenic model involves multiple choices to consider at various steps of the process. Our past experiences with previous transgenic mouse models of SCA10 can guide some of these choices. The value of any transgenic mouse model to mimic the pathological mechanisms of a human disease rests upon its ability to faithfully mimic aspects of the human disease in a lower vertebrate organism.
